# Supplementary material for: Stable endocytic structures navigate the complex pellicle of apicomplexan parasites
Source: Nat Commun. 2023 Apr 15;14:2167. doi: 10.1038/s41467-023-37431-x (PMC10105704; doi:10.1038/s41467-023-37431-x)
Supplement: Supplementary file 1 — Supplementary information [file 41467_2023_37431_MOESM1_ESM.pdf]

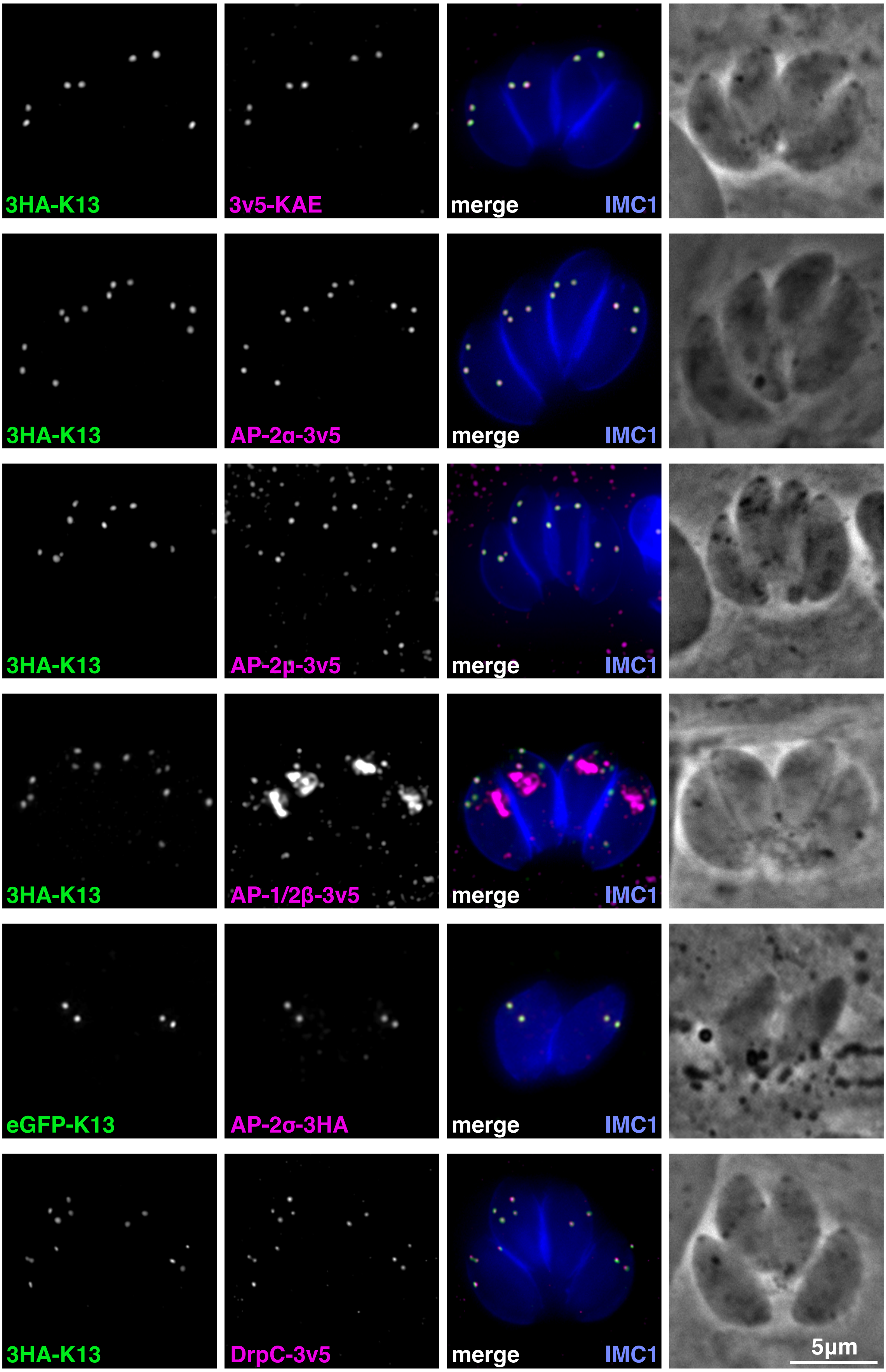

Figure S1: Wide-field fluorescence images of AP-2 subunits, novel AP-2 derived KAE, and DrpC associated with the K13 complex. Only AP1/2β shows additional locations to K13 and this is consistent with it being shared between the AP-2 and the post-Golgi trafficking adaptor AP-1.

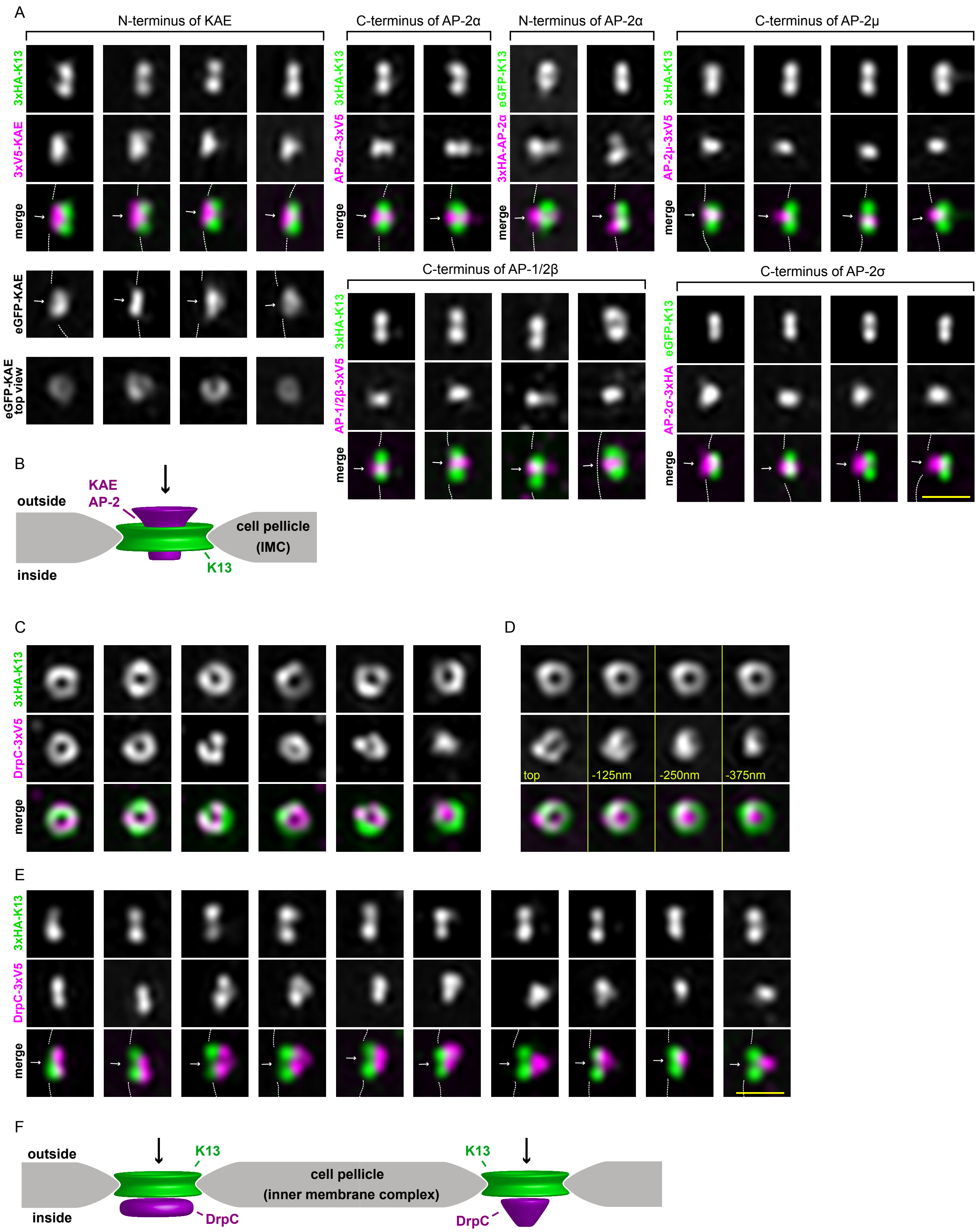

Figure S2: Projections of AP-2, AP-2-derived protein KAE, and DrpC observed by 3D-SIM. (A) Side projections of 3D-SIM images of multiple independent micropores showing K13 and the four AP-2 subunits and the AP-2α-derived protein KAE. Arrows indicate the plasma membrane side of the complex, and cross sections of the top of the complex for AP-2ale shows the annular mouth of this funnel. The scale bar for all is 0.5 μm. (B) Model of the AP-2 complex locations with respect to K13 and the IMC. (C) Multiple examples of micropores seen in top projection showing DrpC states ranging from ring to punctum while K13 rings stay uniform in diameter. (D) Sequence of optical sections of the DrpC signal through an individual K13-DrpC complex. (E) Multiple micropores seen in side projection showing DrpC states compared to K13. Scale bar for all is 0.5 μm. (F) Model of the DrpC locations with respect to K13 and the IMC.

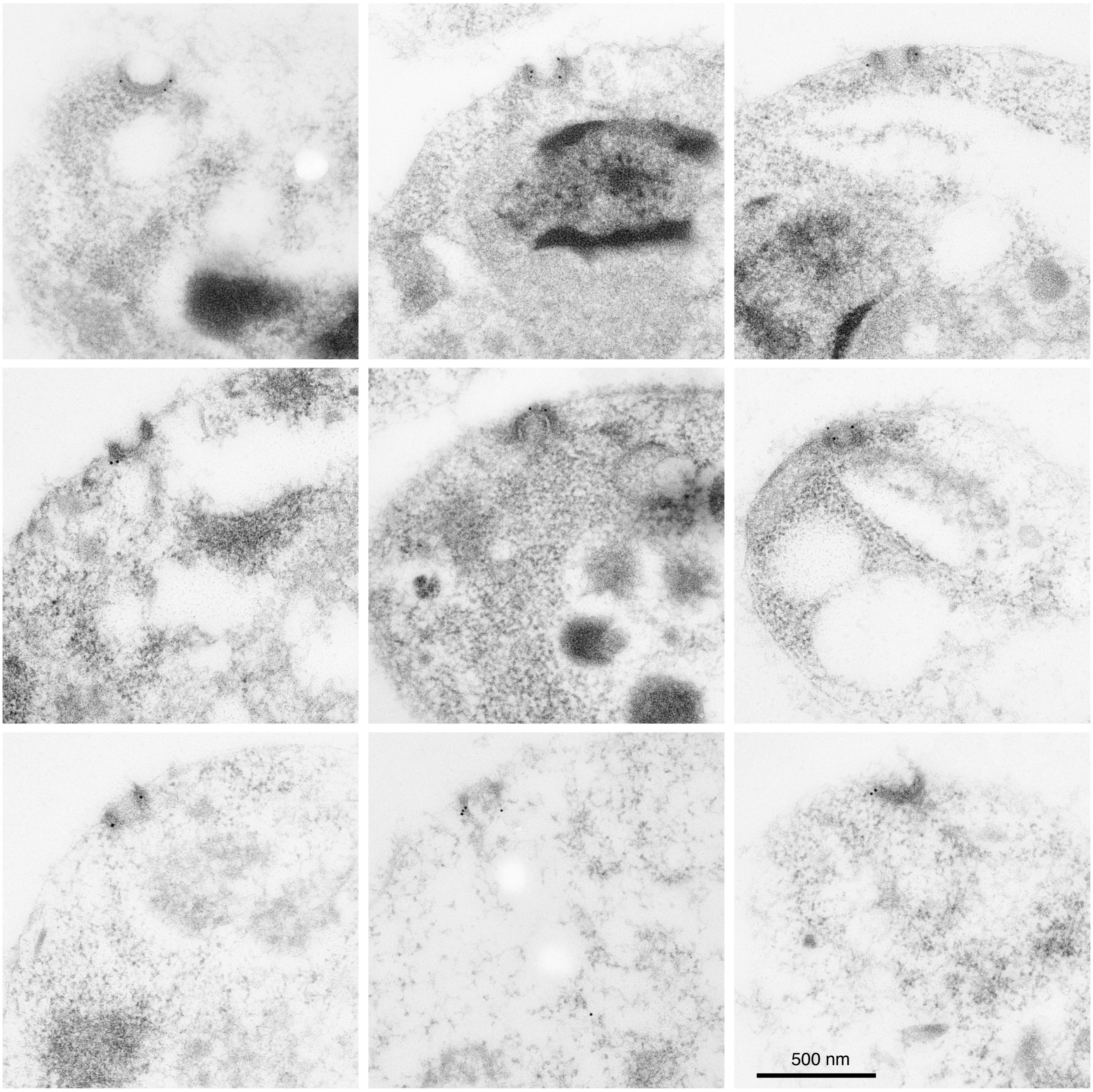

Figure S3: Immuno-gold labelling of 3v5-K13 in *T. gondii* thin sections observed by transmission electron microscopy. Nine examples shown all at the same magnification. Negative controls were performed using the gold-conjugated secondary antibody only and no labelling of the micropore was seen (not shown).

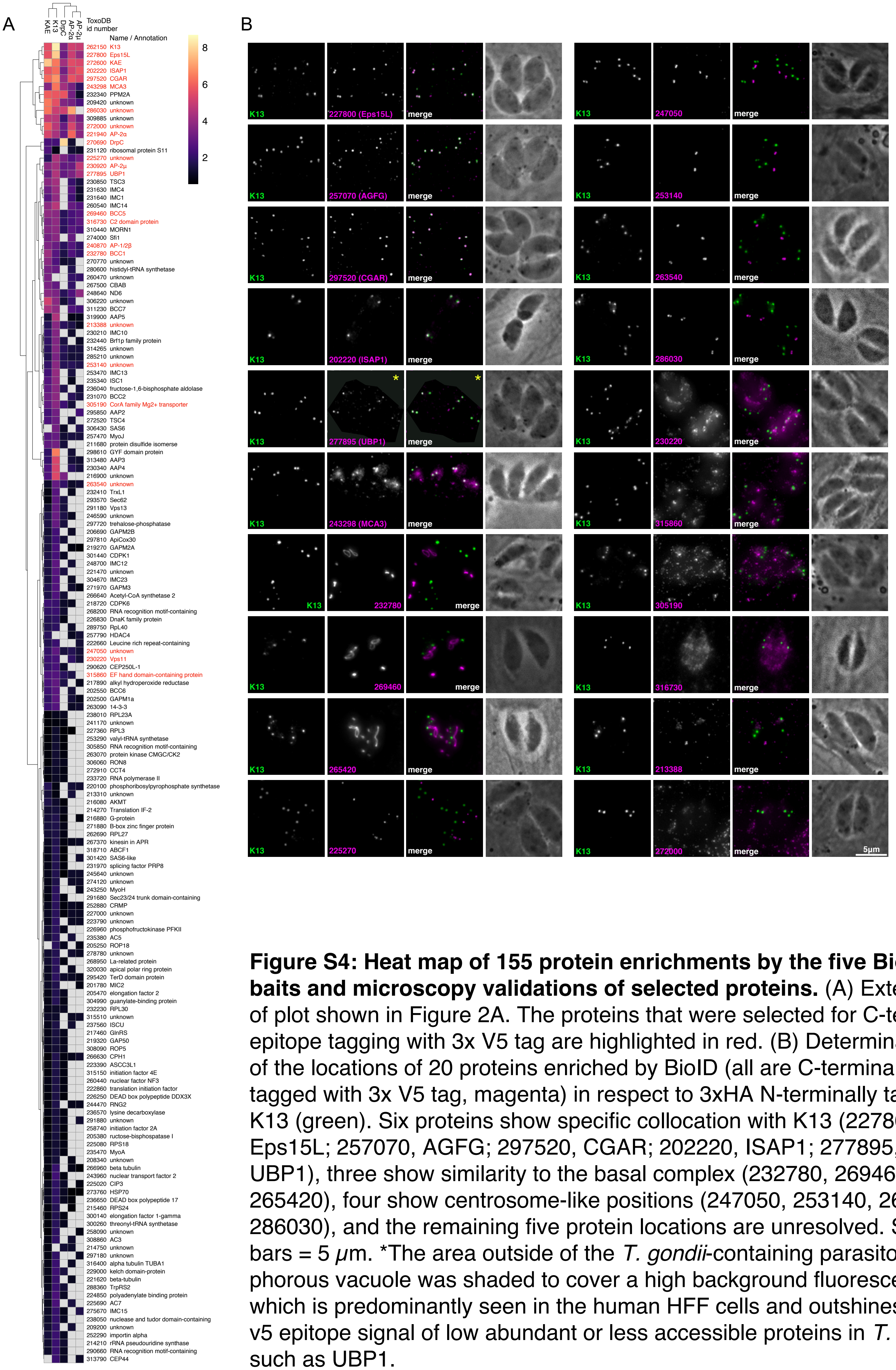

Figure S4: Heat map of 155 protein enrichments by the five BioID baits and microscopy validations of selected proteins. (A) Extension of plot shown in Figure 2A. The proteins that were selected for C-terminal epitope tagging with 3x V5 tag are highlighted in red. (B) Determination of the locations of 20 proteins enriched by BioID (all are C-terminally tagged with 3x V5 tag, magenta) in respect to 3xHA N-terminally tagged K13 (green). Six proteins show specific collocation with K13 (227800, Eps15L; 257070, AGFG; 297520, CGAR; 202220, ISAP1; 277895, UBP1), three show similarity to the basal complex (232780, 269460, 265420), four show centrosome-like positions (247050, 253140, 263540, 286030), and the remaining five protein locations are unresolved. Scale bars = 5 μm. *The area outside of the *T. gondii*-containing parasitophorous vacuole was shaded to cover a high background fluorescence, which is predominantly seen in the human HFF cells and outshines the v5 epitope signal of low abundant or less accessible proteins in *T. gondii* such as UBP1.

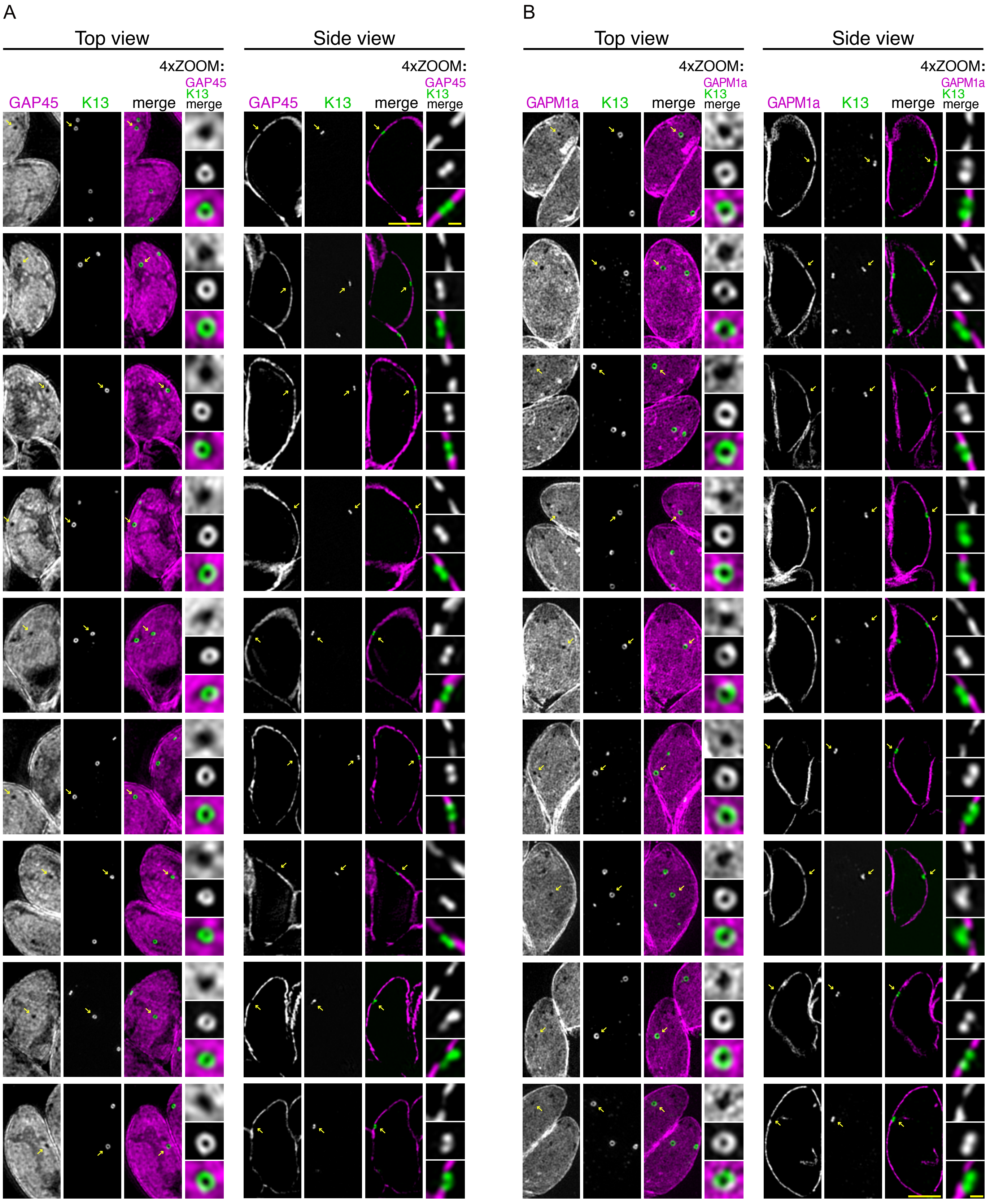

Figure S5: IMC markers GAP45 and GAPM1a are excluded from the sites of the K13 micropore ring. Multiple micropores seen in 3D-SIM images either in top or side view of the structure. K13 is shown in green and GAP45 (A) and GAPM1a (B) are shown in magenta. The yellow arrows point to the zoomed-in area. The scale bars (bottom right corner) are 2 µm for the whole cell view and 0.2 µm for the zoomed-in cropped image.

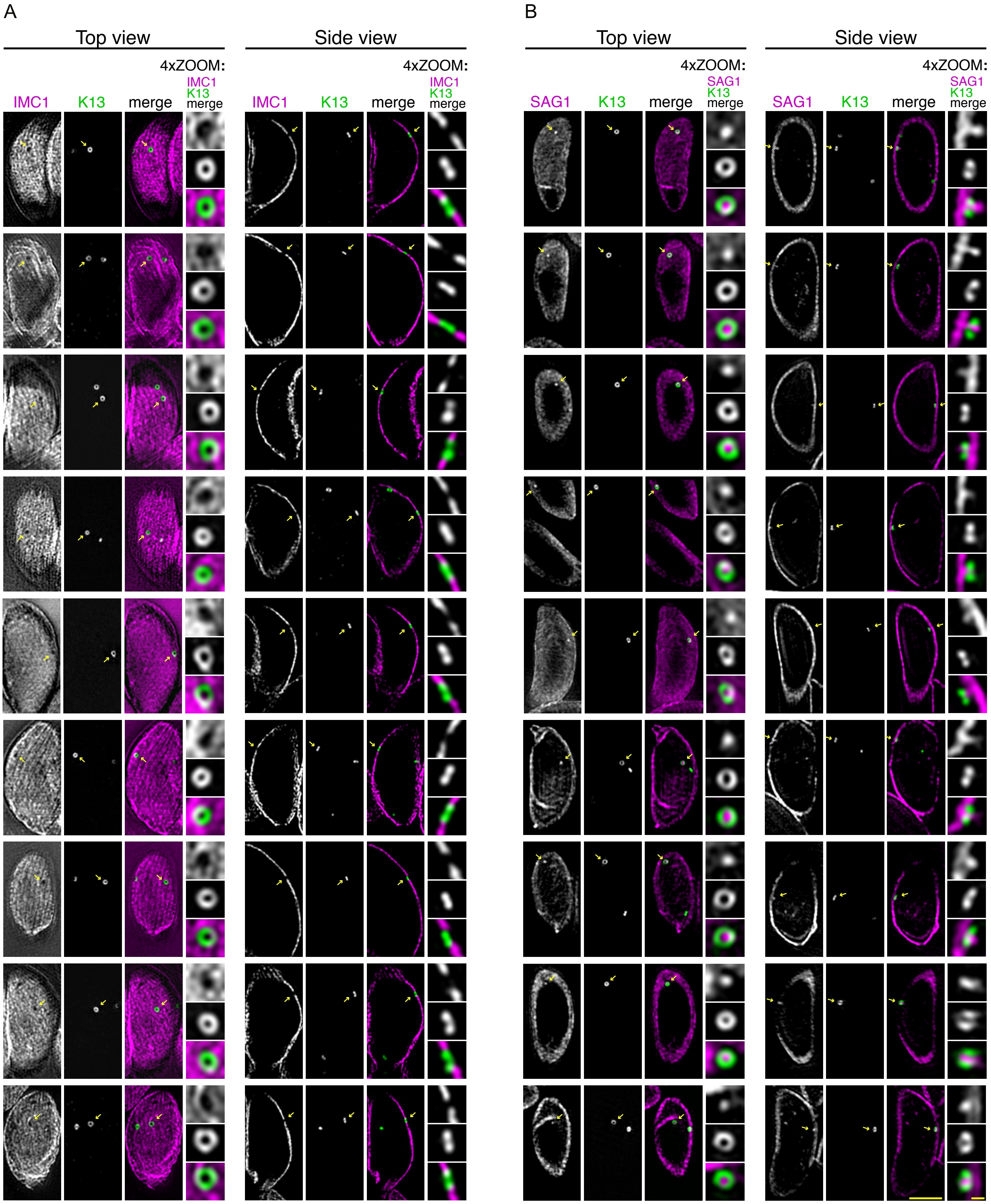

Figure S6: IMC marker IMC1 is excluded from the sites of the K13 micropore ring whereas outer plasma membrane protein SAG1 penetrates the ring as a small invagination. Multiple micropores seen in 3D-SIM images either in top or side view of the structure. K13 is shown in green and IMC1 (A) and SAG1 (B) are shown in magenta. The yellow arrows point to the zoomed-in area. The scale bars (bottom right corner) are 2 µm for the whole cell view and 0.2 µm for the zoomed-in cropped image.

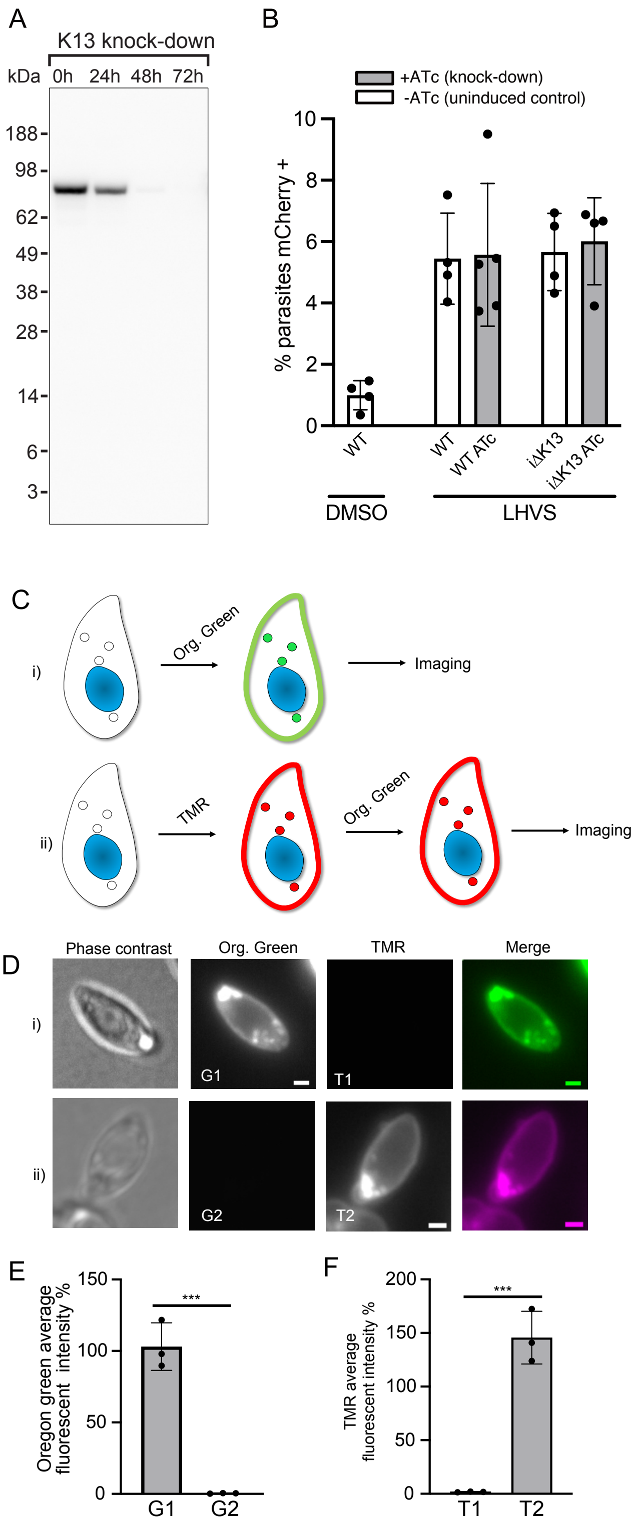

Figure S7: Endocytosis assays. (A) K13 depletion detected by Western blot over 72 hours of ATc-induced mRNA down-regulation. (B) Percentage of parasites with detectable host cytosol-expressed mCherry accumulation with 24 h cathepsin L inhibitor LHVS treatment or DMSO vehicle control. Parental (wild type) or iΔHA-TgK13 cells were given 24 hours to accumulate mCherry following 48 hours plus or minus ATc-treatment. P values for WT DMSO vs WT LHVS, WT LHVS vs WT LHVS ATc, iΔK13 uninduced vs iΔK13 ATc are 0.0012, 0.9290, 0.7239, respectively. (C) Schematic of ligand binding to test for saturability of the SAG1 pools by Halo ligands. (D) i) Membrane-permeable Oregon Green labels both surface and intracellular SAG1 vesicles when used alone. ii) When membrane-permeable TMR biding is applied first, no binding of either external or internal SAG1 pools is seen by subsequent Oregon Green application. Scale bars = 1 μm. (E,F) Quantitation of parasite fluorescence for treatments according to (D), P-values are indicated as $0 \leq P \leq 0.001$, *** and the exact values are 1.83E-16 for (E) and 4.55E-11 for (F). Three (B) or four (E,F) biological replicates were used for each analysis; the error bars are standard deviations and the centre is mean. Two-sided Student's T test was used for all the comparisons with no adjustments. Source data are provided as Source Data file.

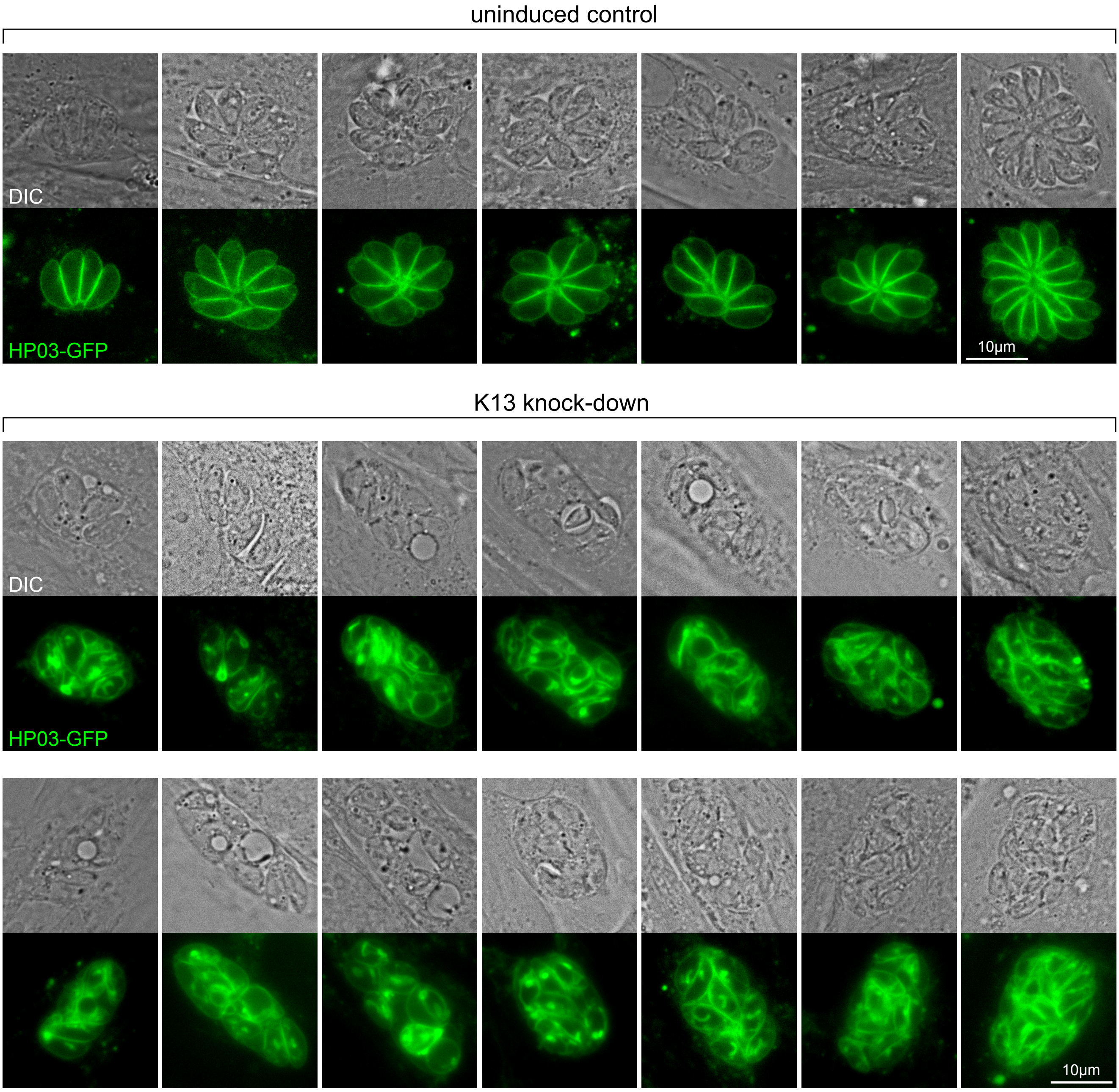

Figure S8: Plasma membrane and parasite rosette disruption upon K13 depletion. Live-cell microscopy by Differential Interference Contrast (DIC) and fluorescence detection of integral plasma membrane protein marker HP03-GFP shows loss of parasite organization and excess of plasma membrane in these further examples from Fig 6F.

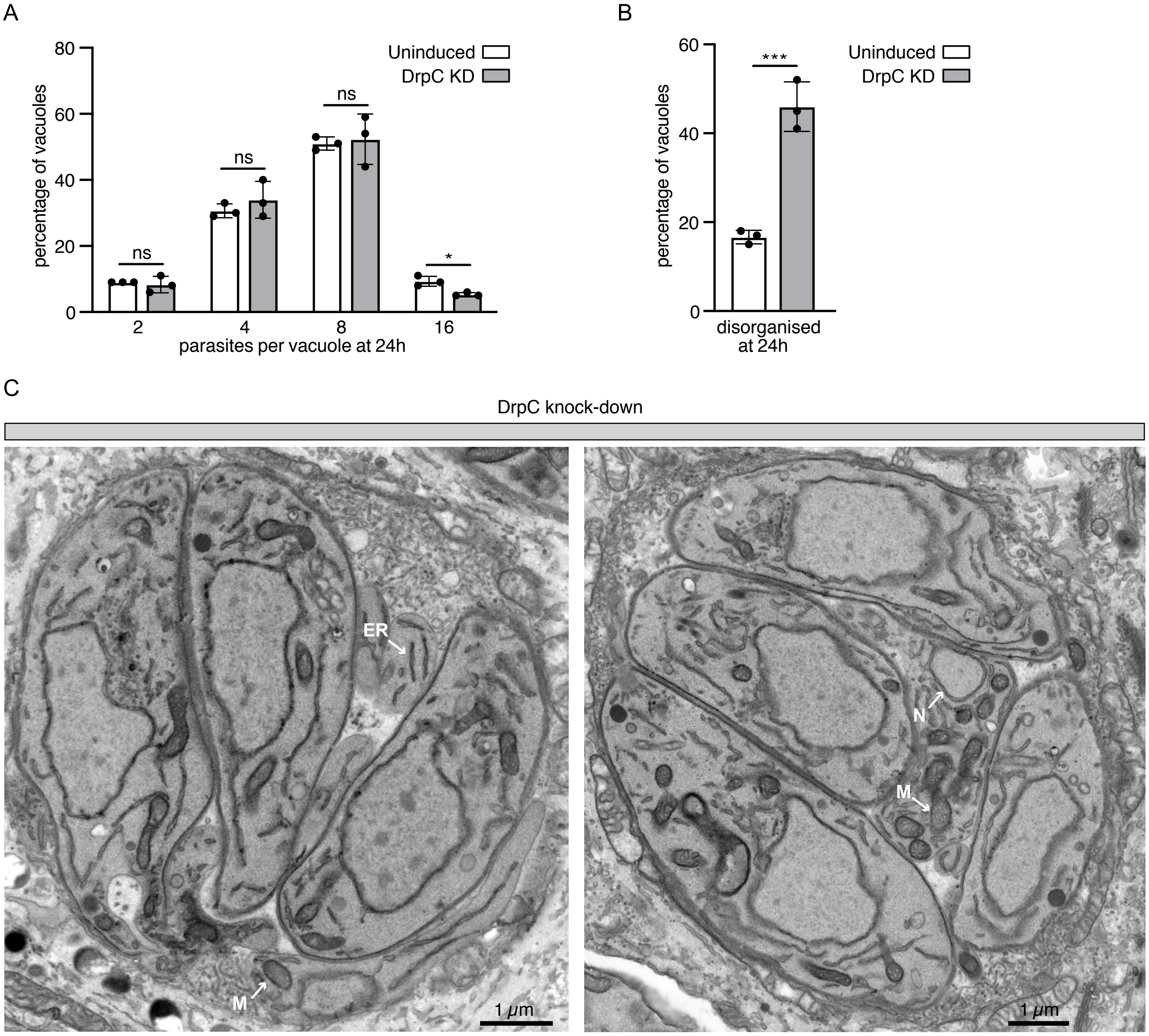

Figure S9: DrpC depletion phenotypes. Depletion of DrpC for 72 hours (48 hours prior to inoculation of new host cells and a further 24 hours of growth) results in (A) no change in replication rate (P values: 2 parasites: 0.6702, 4 parasites: 0.3864, 8 parasites: 0.7844, 16 parasites: 0.0132), but (B) increased frequency of disordered vacuoles (P value=0.0009) and (C) atypical extensions of the plasma membrane-bound cytosol between parasites. ER, endoplasmic reticulum; N, nucleus; M, mitochondrion; Scale bars = 1 μm. Three biological replicates were used for each analysis. P-values are indicated as $0.01<P\leq 1$, ns; $0.01<P\leq 0.05$, *; $0\leq P\leq 0.001$, ***, error bars are standard deviations and the centre measurement of graph bars is mean. Two-sided Student's T test was used for all the comparisons with no adjustments. Source data are provided as Source Data file.

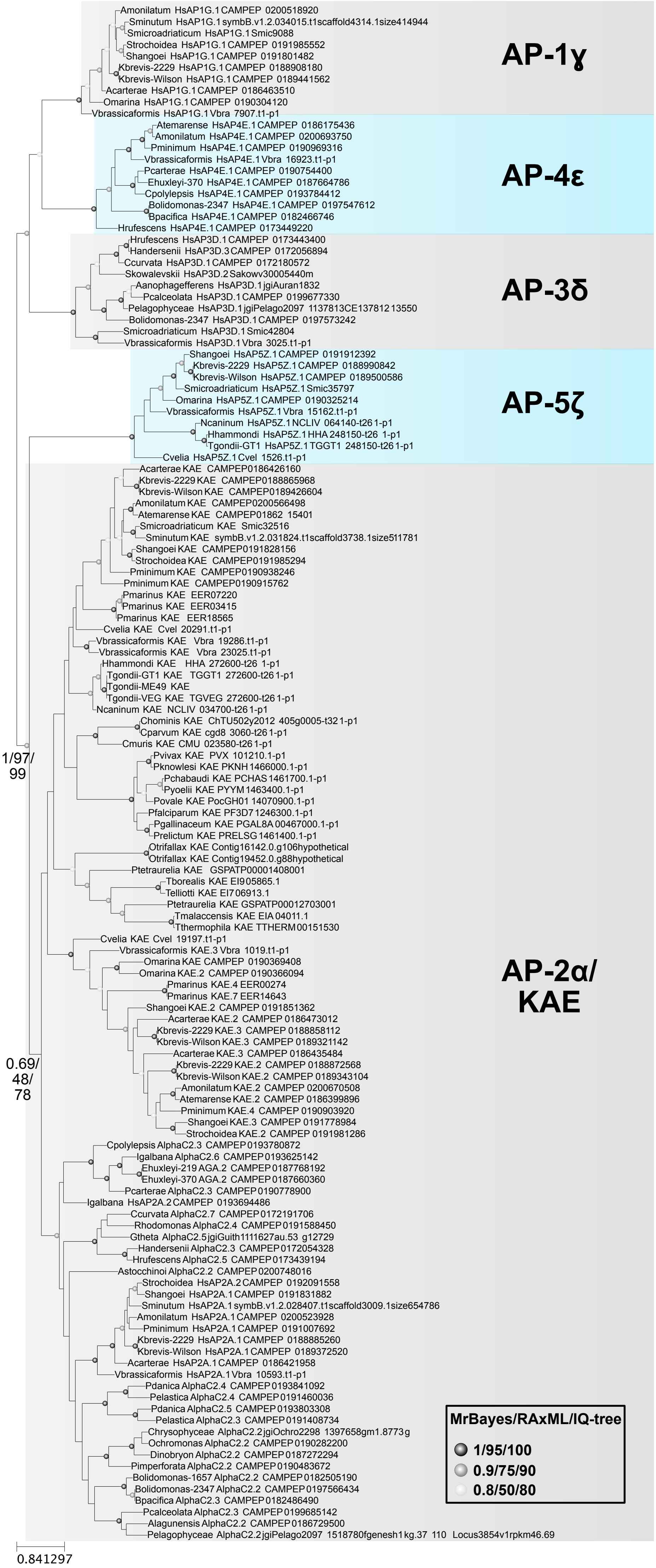

Figure S10: Maximum likelihood phylogeny of AP ear domains showing that KAE is derived from a duplication of AP-2α. KAE is only found in alveolate taxa and only dinoflagellates, perkinsids and chrompodellids maintain a second common KAE paralogue. Branch supports were evaluated based on MrBayes posterior probabilities and bootstrap analyses in RAxML and IQ-tree.

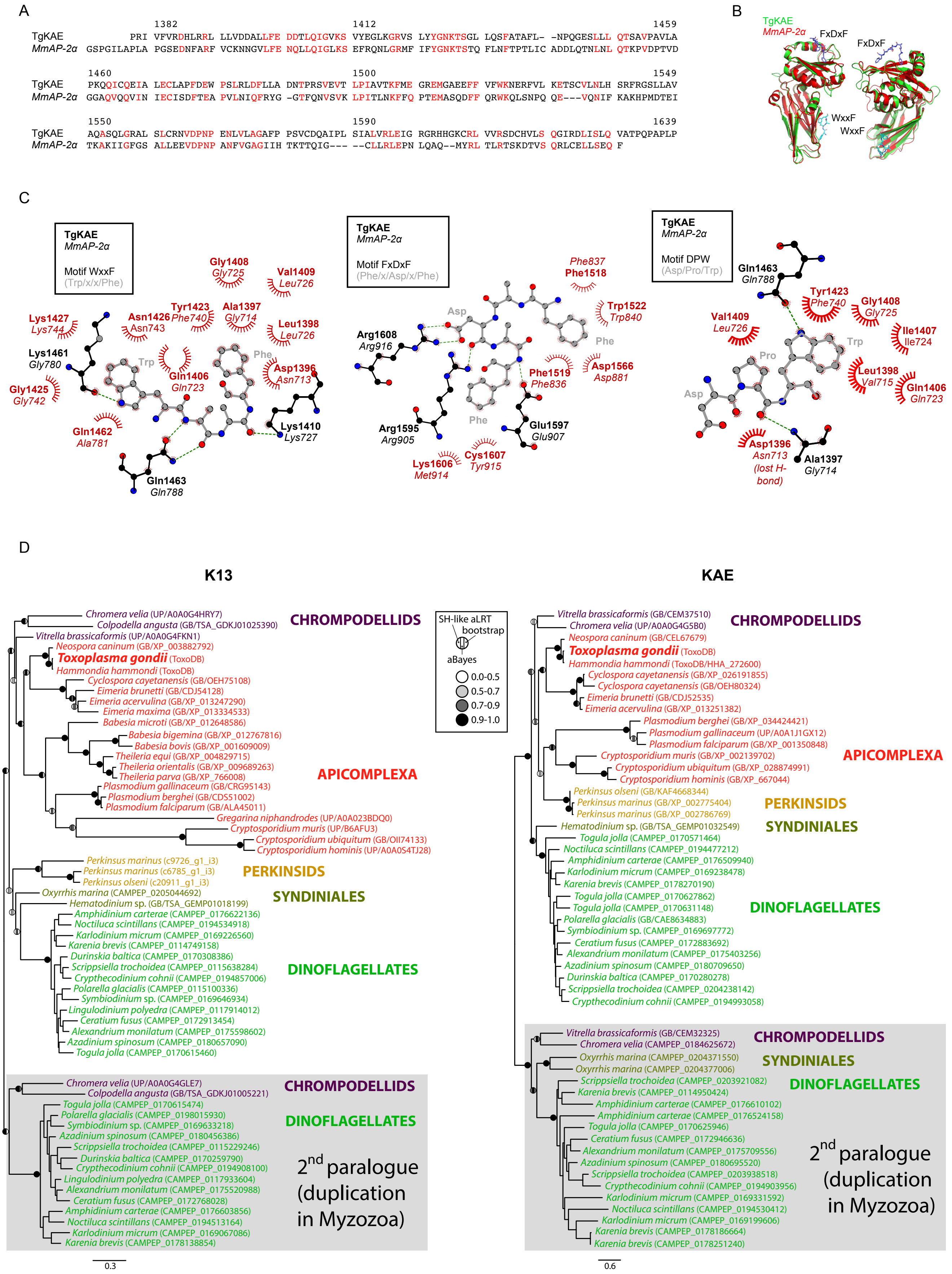

Figure S11: Structural and evolutionary analysis of KAE and K13. Comparison of TgKAE and MmAP-2α by (A) Sequence alignments of TgKAE and MmAP-2α ear domains (31% identity (red), 47% positive) (numbering is for TgKAE), and (B) modelling based on the MmAP-2α structure (PDB 1w80) indicate a strong structural conservation. The positions of FxDxF and WxxF Eps15 binding sites are shown. (C) The TgKAE model indicates that the binding sites for FxDxF, DPW and WxxF motifs are likely conserved (equivalent residues in MmAP-2α are shown in italics for comparison). The peptide motif and TgKAE side chains are shown in ball-and-stick representation, with carbon colored grey and black respectively, oxygen in red and nitrogen in blue. Hydrogen bonds are shown as green dotted lines, while the spoked arcs represent protein residues making nonbonded contacts with the peptide motif. (D) Maximum likelihood phylogenies of K13 and KAE. The ML tree topology and branch supports (SH-like aLRT, aBayes and bootstrap, respectively) were calculated in PhyML 3.1.
