# Supplementary material for: Stable endocytic structures navigate the complex pellicle of apicomplexan parasites
Source: Nat Commun. 2023 Apr 15;14:2167. doi: 10.1038/s41467-023-37431-x (PMC10105704; doi:10.1038/s41467-023-37431-x)
Supplement: Supplementary file 2 — Description of additional supplementary files [file 41467_2023_37431_MOESM2_ESM.docx]

**Description of additional supplementary files**

Title: Supplementary data 1

Description: BioID peptide and protein identification and quantification data

Title: Supplementary data 2

Description: BioID protein enrichments by bait protein

Title: Supplementary data 3

Description: Oligonucleotides and plasmids used for genetic modifications
