# Supplementary material for: Stable endocytic structures navigate the complex pellicle of apicomplexan parasites
Source: Nat Commun. 2023 Apr 15;14:2167. doi: 10.1038/s41467-023-37431-x (PMC10105704; doi:10.1038/s41467-023-37431-x)
Supplement: Supplementary file 6 — Reporting Summary [file 41467_2023_37431_MOESM6_ESM.pdf]

nature portfolio

Corresponding author(s): Ross Waller, Simon Gras
Last updated by author(s): Mar 15, 2023

Reporting Summary

Nature Portfolio wishes to improve the reproducibility of the work that we publish. This form provides structure for consistency and transparency in reporting. For further information on Nature Portfolio policies, see our Editorial Policies and the Editorial Policy Checklist.

Statistics

For all statistical analyses, confirm that the following items are present in the figure legend, table legend, main text, or Methods section.

n/a	Confirmed	
☐	☒	The exact sample size (*n*) for each experimental group/condition, given as a discrete number and unit of measurement
☐	☒	A statement on whether measurements were taken from distinct samples or whether the same sample was measured repeatedly
☐	☒	The statistical test(s) used AND whether they are one- or two-sided<br>*Only common tests should be described solely by name; describe more complex techniques in the Methods section.*
☐	☒	A description of all covariates tested
☐	☒	A description of any assumptions or corrections, such as tests of normality and adjustment for multiple comparisons
☐	☒	A full description of the statistical parameters including central tendency (e.g. means) or other basic estimates (e.g. regression coefficient) AND variation (e.g. standard deviation) or associated estimates of uncertainty (e.g. confidence intervals)
☐	☒	For null hypothesis testing, the test statistic (e.g. *F*, *t*, *r*) with confidence intervals, effect sizes, degrees of freedom and *P* value noted<br>*Give P values as exact values whenever suitable.*
☐	☒	For Bayesian analysis, information on the choice of priors and Markov chain Monte Carlo settings
☒	☐	For hierarchical and complex designs, identification of the appropriate level for tests and full reporting of outcomes
☒	☐	Estimates of effect sizes (e.g. Cohen's *d*, Pearson's *r*), indicating how they were calculated

Our web collection on statistics for biologists contains articles on many of the points above.

Software and code

Policy information about availability of computer code

Data collection	Raw LC-MS data were recorded, managed, and visualised in Xcalibur v3.0.63. Peptide-spectrum matching was performed with Mascot v2.6.2. Percolator v3.02.1 was used for the validation of the false discovery rate of peptide-spectrum matches. Proteome Discoverer v2.3 was used for raw mass spectra processing and TMT reporter ion quantification, peptide and protein grouping, peptide- and protein-level FDR control.
Data analysis	BioID data analysis was performed with R v3.6 using packages tidyverse v1.2.1 and pheatmap v1.0.12, and Bioconductor packages MSnbase v2.10.1, biobroom v1.16.0, and limma v3.40.6. We used STATA v.14. for statistical analyses and the graphs were done using Prism v8. We used SWISS-MODEL (swissmodel.expasy.org) and coot v1 (www2.mrc-lmb.cam.ac.uk/personal/pemsley/coot) for protein 3D structure modeling and DomPRED (http://bioinf.cs.ucl.ac.uk/software.html) was used for protein domain-boundaries prediction. Phylogenetic data were analysed at the CIPRES online portal (www.phylo.org/) or locally using RAxML v8.2.1232, IQ-TREE v1.6.12, PhyML-3.1 and MrBayes v3.2.7a35. The structured illumination images were reconstructed in softWoRx v6.1.3 (Applied Precision). All fluorescence images were processed using ImageJ v1.53.

For manuscripts utilizing custom algorithms or software that are central to the research but not yet described in published literature, software must be made available to editors and reviewers. We strongly encourage code deposition in a community repository (e.g. GitHub). See the Nature Portfolio guidelines for submitting code & software for further information.

Data

Policy information about availability of data

All manuscripts must include a data availability statement. This statement should provide the following information, where applicable:

- Accession codes, unique identifiers, or web links for publicly available datasets
- A description of any restrictions on data availability
- For clinical datasets or third party data, please ensure that the statement adheres to our policy

The mass spectrometry proteomics data have been deposited to the ProteomeXchange Consortium via the PRIDE partner repository with the dataset identifier PXD034193. ToxoDB accession numbers of the BioID hits and the localised proteins are shown directly in the corresponding figures (Fig 2, Fig S4). The sequence identifiers for the protein sequences that were used for phylogenetic analyses are shown for each taxon in the phylogenetic trees (Fig S10, S11). The structure of the mouse Alpha-adaptin Appendage Domain (identifier 1w80) was retrieved from the Protein Data Bank (PDB).

Human research participants

Policy information about studies involving human research participants and Sex and Gender in Research.

Reporting on sex and gender	N/A
Population characteristics	N/A
Recruitment	N/A
Ethics oversight	N/A

Note that full information on the approval of the study protocol must also be provided in the manuscript.

Field-specific reporting

Please select the one below that is the best fit for your research. If you are not sure, read the appropriate sections before making your selection.

[x] Life sciences [ ] Behavioural & social sciences [ ] Ecological, evolutionary & environmental sciences

For a reference copy of the document with all sections, see nature.com/documents/nr-reporting-summary-flat.pdf

Life sciences study design

All studies must disclose on these points even when the disclosure is negative.

Sample size

No sample size determination was done in the case of BioID experiments. Instead, 3 biological replicates were analysed as the minimum number of replicates required for the inference of the mean and variance of protein abundance values measured for each sample group, which is a standard practice for this type of experiments in the field.
We used an independent proportion comparison method to determine the minimal sample size for the replication, egress, endocytosis and plasma membrane and vacuole organisation assays. The minimum sample size required to find a difference of proportions of at least 40% between the negative control uninduced cell lines (NC) and the knock down (KD) cell lines was 40 vacuoles/parasites per replicate (20 KD + 20 NC). The numbers used for each experiment (~200 vacuoles per condition) for these assays were well above the recommended minimum. Each of the aforementioned assays was performed 3 times independently, which is a standard practice for this type of experiments in the field.

Data exclusions

In the BioID data, only proteins with High and Medium Protein FDR Confidence levels ($q \leq 0.05$) with complete abundance value series across 3 Bait and 3 NC samples were considered for the statistical analysis. The proteins with missing values in the TMT quantification data were considered separately on a case-by-case basis, but no statistical testing was applied.

Replication

All replicates of the BioID experiments obtained successfully generated sufficient numbers of cells and afforded sufficient amounts of proteins and peptides for the subsequent LC-MS/MS analysis. All replicates of the replication, egress, endocytosis and plasma membrane and vacuole organisation assays were successful and enough vacuoles/parasites were obtained for a robust statistical analysis.

Randomization

For the BioID experiments, three TMT10plex sets were used. The samples were distributed as follows:
negative control: Set 1 - TMT126, TMT127N, TMT127C; Set 2 - TMT127C, TMT128N, TMT128C; Set 3 - TMT128C, TMT129N, TMT129C
K13: Set 1 - TMT128N, TMT128C, TMT129N
DrpC: Set 1 - TMT129C, TMT130N, TMT130C
AP2-alpha: Set 2 - TMT126, TMT127N, TMT129N
AP2-mu: Set 3 - TMT130N, TMT130C, TMT131
KAE: Set 2 - TMT130N, TMT130C, TMT131
The remaining available TMT10plex channels were used to label samples from an unrelated experiment. All samples were labelled on the same day using freshly prepared TMT10plex reagent solutions and according to the manufacturer's protocol ensuring the high efficiency of

TMT-labelling, which deemed sample randomisation within each set unnecessary. To account for the TMT ratio compression due to isotopic impurity of the TMT10plex labelling reagents, manufacturer-provided correction factors were applied to the measured TMT10plex reporter ion intensities.
The parasites/vacuoles that were counted for each experiments of the replication, egress, endocytosis and plasma membrane and vacuole organisation assays were selected randomly. Specifically, several fields of view were obtained for each sample by shifting the position of the sample slide under the microscope in one direction until enough parasites/vaculoes were counted. For each randomly selected field of view all of the parasites/vacuoles were counted to avoid biases.

Blinding

The acquisition of LC-MS/MS data was performed at a core facility, the Cambridge Centre for Proteomics, by a facility staff scientist who was not involved in the design of this study and was blinded to the study factors and sample identity. Blinding was not performed for the replication, egress, endocytosis and plasma membrane and vacuole organisation assays as the scored characteristics were clearly and easily distinguishable and the risk of potential bias was therefore minimal.

Reporting for specific materials, systems and methods

We require information from authors about some types of materials, experimental systems and methods used in many studies. Here, indicate whether each material, system or method listed is relevant to your study. If you are not sure if a list item applies to your research, read the appropriate section before selecting a response.

Materials & experimental systems

n/a	Involved in the study
☐	☒ Antibodies
☐	☒ Eukaryotic cell lines
☒	☐ Palaeontology and archaeology
☒	☐ Animals and other organisms
☒	☐ Clinical data
☒	☐ Dual use research of concern

Methods

n/a	Involved in the study
☒	☐ ChIP-seq
☒	☐ Flow cytometry
☒	☐ MRI-based neuroimaging

Antibodies

Antibodies used

Primary antibodies: anti-HA High Affinity (ROAHAHA, Roche 11867423001), anti-V5 (ThermoFisher R960-25), anti-SAG1 (ThermoFisher MA5-18268), anti-GAP45 & anti-IMC1 (both gift from Dominique Soldati-Favre, University of Geneva, Switzerland), anti-GST (Invitrogen 700775).
Secondary antibodies: Goat anti-mouse Alexa Fluor 488 (ThermoFisher A-11029), Goat anti-rabbit Alexa Fluor 594 (ThermoFisher A-11012), Goat anti-rat Alexa Fluor 594 (ThermoFisher A-11007), Goat anti-rabbit Alexa Fluor 488 (ThermoFisher A-11008), Goat anti-rabbit Alexa Fluor 405 (ThermoFisher A-31556), Goat anti-mouse IgG H&L (10nm Gold) (Abcam ab39619), Goat anti-rabbit IgG (H +L) Secondary Antibody, HRP

Validation

anti-HA High Affinity (ROAHAHA, Roche 11867423001) was validated by the manufacturer by western blot analysis; anti-V5 (ThermoFisher R960-25) was verified by the manufacturer by Relative expression to ensure that the antibody binds to the antigen stated; anti-SAG1 (ThermoFisher MA5-18268) was validated by western blot analysis by Holmes et al. (PLoS Pathog. 2021. 17:e1009335); anti-GST (Invitrogen 700775) antibody specificity was demonstrated by the manufacturer by detection of different targets fused to GST tag in transiently transfected lysates tested. Relative detection of GST tag was observed across different proteins fused with GST tag; anti-IMC1 was validated by western blot analysis and immunofluorescence assay (IFA) by Mann and Beckers (Molecular & Biochemical Parasitology 2001. 115:257–268); anti-GAP45 was validated by western blot analysis and IFA by Plattner et al. (Cell Host & Microbe 2008. 3:77-87) and Frénal et al. (Cell Host & Microbe 2010. 8:343–357).

Eukaryotic cell lines

Policy information about cell lines and Sex and Gender in Research

Cell line source(s)

The Toxoplasma RH strain is widely used in the community of Toxoplasma biologists and freely shared. The host cell HFF (SCRC-1041) and HeLa (CCL-2) cell lines are commercially available from ATCC. Sources of RH Δku80/TATi and RH Δku80/Tir1 are referenced in the manuscript.

Authentication

All cell lines were authenticated by microscopy and the cell lines with specific genetic modifications were validated by western blot or diagnostic PCR test

Mycoplasma contamination

All cell-lines tested negative for Mycoplasma contamination

Commonly misidentified lines
(See ICLAC register)

No commonly misidentified cell lines were used in this study.
